# Detection of *Leishmania donovani* Status in Dogs (*Canis familiaris,* Linnaeus, 1758) in the Karamoja Subregion of Uganda

**DOI:** 10.1155/jotm/7530351

**Published:** 2025-09-29

**Authors:** Charles D. Kato, Angella Musewa, Tequiero A. Okumu, Margaret Mbuchi, Edwinah Atusingwize, Ivan Ankunda, Santiago C. Tomas, Gloria Pol Ferrer, Benard Matovu, Emmanuel Muhumuza, Marta Planellas Bachs, Jesus Muro Figueres, James Robert Ochieng

**Affiliations:** ^1^Department of Biomolecular Resources and Biolab Sciences, College of Veterinary Medicine, Animal Resources and Biosecurity, Makerere University, P.O. Box 7062, Kampala, Uganda; ^2^Africa One Health University Network, Country Office Kampala, Kampala, Uganda; ^3^Department of Clinical Studies, College of Health Sciences, University of Nairobi, P.O. Box 30197, Nairobi, Kenya; ^4^Kenya Medical Research Institute, P.O. Box 54840, Nairobi, Kenya; ^5^Department of Disease Control and Environmental Health, School of Public Health, Makerere University, P.O. Box 7062, Kampala, Uganda; ^6^Vector Control Division, Ministry of Health, P.O. Box 1661, Kampala, Uganda; ^7^LETI Pharma, Barcelona, Spain; ^8^Department of Zoology, Entomology and Fisheries Sciences, College of Natural Sciences, Makerere University, P.O. Box 7062, Kampala, Uganda; ^9^Department of Biochemistry and Systems Biology, College of Natural Sciences, Makerere University, P.O. Box 7062, Kampala, Uganda; ^10^Department of Animal Medicine and Surgery, Universitat Autònoma de Barcelona, Bellaterra, Spain; ^11^Daktari East Africa, Kampala, Uganda

**Keywords:** dog, Karamoja subregion, *Leishmania donovani*, Uganda

## Abstract

To date, the reservoir host for visceral leishmaniasis, a neglected tropical disease caused by *Leishmania donovani*, is unknown, although studies pointing to dogs, domestic animals, and rodents are emerging. We aimed to investigate whether the dog (*Canis familiaris*, Linnaeus, 1758) is a potential reservoir for *L*. *donovan*i in the Karamoja subregion of Uganda. Blood and lymph node aspirates were purposively collected from dogs (*n* = 139) in disease endemic villages of Amudat and Moroto districts in August 2023. An indirect enzyme-linked immunosorbent assay was used to detect anti-*Leishmania* IgG antibodies in serum. DNA extracted from lymph node aspirates was subjected to a polymerase chain reaction (PCR) targeting the rRNA internal transcribed spacer region of *Leishmania* species. The sera from 139 dogs did not demonstrate any evidence of circulating antibodies against *Leishmania*, as the optical density (OD) values were all below 0.25, lower than the threshold value of 0.45. Similarly, all the dog lymph node DNAs (*n* = 139) were negative for *Leishmania* parasites. Although our results found no evidence to support dogs as reservoirs for *L*. *donovani*, in this study, further research utilizing larger sample sizes is recommended to confirm this finding. Furthermore, the presence of *L*. *donovani* in sand flies and other suspected reservoirs, such as domestic animals and wild rodents, requires further investigation.

## 1. Introduction

Leishmaniasis is a neglected tropical disease that globally affects about 700,000 to 1 million people, with an estimated death toll of around 40,000 annually [1–3]. Visceral leishmaniasis (VL) is the most serious, almost always fatal without treatment [2, 3]. Around 90% of VL global cases are dominated by Kenya, Uganda, India, Brazil, Bangladesh, Ethiopia, Sudan, and South Sudan [3, 4]. Despite the instauration of several control strategies, VL has remained endemic in rural hard-to-reach communities of the Karamoja subregion, Uganda, Turkana, West Pokot, Baringo, Rift Valley, and the western region in Kenya [1, 4]. In 2022 and 2023, Uganda reported 264 and 195 cases, and Kenya reported 1573 and 1252 cases, respectively [5]. The two main parasite species responsible for human infection are *L*. *donovani,* whose transmission is mostly anthroponotic, from human to human [1, 6], and *L*. *infantum*, whose transmission is mostly zoonotic, from animals to humans, with dogs and rodents serving as reservoir hosts [1].

Recently, epidemiological reports from the Indian subcontinent have identified evidence of *L. donovani* infection or exposure to multiple domestic mammals [7]. In Ethiopia, *L. donovani* has been isolated from wild rodent samples [8], and serological evidence of *L. donovani* infection was detected in healthy dogs [9]. Similarly, in neighboring Sudan, antibodies to *L. donovani* were detected in rats, goats, and cows [10]. In Kenya, isolates of *Leishmania* parasites from dogs were reported, with one of the cases adjacent to VL endemic foci in Uganda [11]; however, no other studies were conducted in the region to follow it up. In the Karamoja subregion, Uganda, most of the population is seminomadic pastoralists who keep dogs mostly for guarding property, including livestock, herding, and hunting. These dogs roam freely in communities, and if they are reservoirs for *Leishmania* species, they could increase the risk of parasite transmission among wildlife, livestock, and humans [12]. Despite dogs being known as reservoirs in other regions, scant information exists on the possibility of a zoonotic component of *L*. *donovani* in the Karamoja subregion of Uganda. This study aimed to investigate whether the dog (*Canis familiaris, Linnaeus*, 1758) is a potential reservoir for *L*. *donovani* in the Karamoja subregion of Uganda.

## 2. Materials and Methods

### 2.1. Study Design

**Figure 1:**
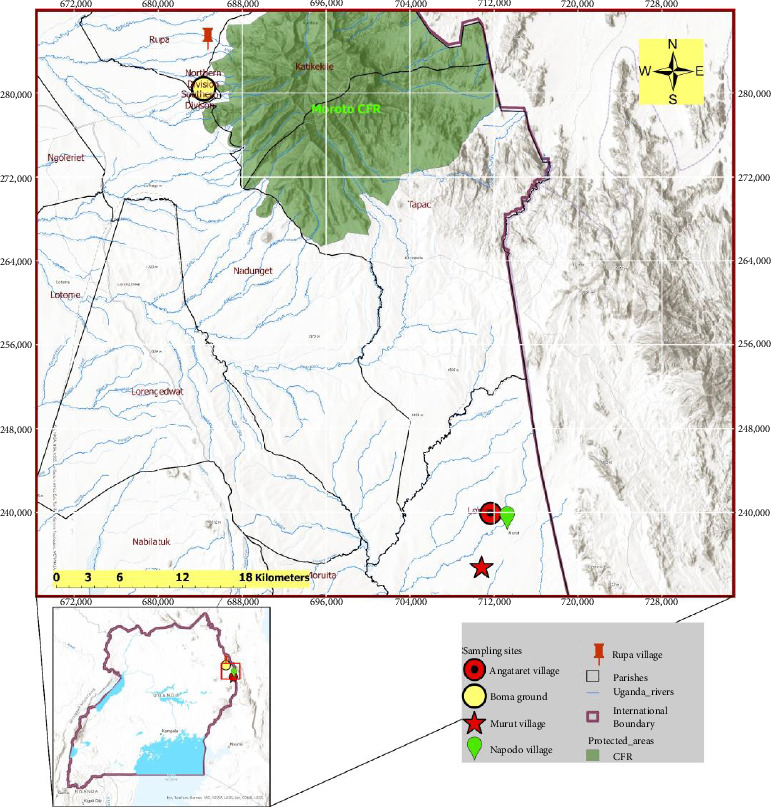
Map showing the dog *Leishmania* active screening sites in the Karamoja subregion, Uganda. Dog sampling was conducted in five villages: Rupa, Boma, Murut, Napodo, and Angataret.

We conducted a cross-sectional study that involved sampling domestic dogs in the Moroto and Amudat districts within the Karamoja subregion of Uganda. The Karamoja subregion is semiarid and is economically the poorest region in Uganda, with the population living on less than US$1.3 and one or no meal per day, increasing vulnerability to leishmaniasis and other diseases [12, 13]. The Karamoja subregion communities are tribal, seminomadic pastoralists [14, 15]. In this current study, we purposively carried out active dog screening [16] in five hotspot villages: Rupa and Boma ground in Moroto District, and Murut, Napodo, and Angataret in Amudat District (Figure 1). The hotspot villages were selected following VL clinical records from 2017 to 2022 at the referral hospitals in Moroto and Amudat districts.

### 2.2. Sample Collection From Dogs

Through the help of the Moroto and Amudat District veterinary offices and village health teams (VHTs), dog owners were sensitized and mobilized to bring their dogs to the respective sites on scheduled days in August 2023. Following the recommended veterinary procedures [17], and with the help of a rope and a Baskerville muzzle tied around the dog's mouth, under the legs of the owner, 139 dogs were sampled. A 5-mL blood sample was collected from the cephalic vein of each dog into a plain vacutainer for ELISA screening [16]. Then, the prescapular lymph node area was quickly shaved and disinfected. The lymph node cytological sample cells were obtained by puncture and then suspended with serum in an EDTA tube [18]. The aliquots for ELISA were spun immediately after every field collection at 3500 rpm for 15 min and kept in 1.8-mL cryotubes, then stored at −196°C in liquid nitrogen until laboratory diagnosis.

### 2.3. Detection of *Leishmania* Antibodies

Leishmaniasis antibodies in the collected dog sera were examined using an indirect ELISA, following the protocol of a commercial kit (INGEZIM LEISHMANIA 15.LSH.K1, Ingenasa, Spain: https://www.diachel.gr/ingenasa-es-ingezim-leishmania-15.lsh.k.1-el; https://www.goldstandarddiagnostics.com/pub/media/productattachments/files/15LSHK1_Technical_sheet_leishmania.pdf) according to the manufacturer's instructions, as described previously by Ledesma et al. [19] and Rodriguez-Cortes et al. [20]. Briefly, positive, serum-free negative control, and dog sera samples were added accordingly. Microtiter immunoassay plates (Greiner, Frickenhausen, Germany) were coated with total soluble *L*. *infantum* promastigote antigens in 100 μL of coating buffer for 18 h at 4°C in the dark. Free binding sites were blocked with a 2% casein solution for 1 h at 37°C. After three washes with 1XPBS-Tween (0.05%), plates were incubated with 100 μL of canine sera for 1 h at 37°C. Plates were washed three times with 1XPBS and incubated with 1:1000-fold diluted anticanine IgG antibody (Sigma, USA), horseradish peroxidase-conjugate. The optical density (OD) was measured at 450 nm in an ELISA microplate spectrophotometer (SpectraMax Plus, USA) within 5 min after the addition of stop solution.

### 2.4. DNA Extraction From Dog Lymph Node Samples

Dog lymph node DNA was extracted using the PureLink spin column kit (Thermo Fisher Scientific, USA) as described by the manufacturer. This involved adding 200 μL of lymph node samples to a sterile 2-mL microcentrifuge tube, followed by 20 μL of Proteinase K and 20 μL of RNase A. About 200 μL of PureLink Genomic Lysis/Binding Buffer was also added, and vortexed briefly for homogenous mixing. The sample mixture was incubated at 55°C for 30 min with brief vortexing at 10-min intervals to encourage complete lysis of cells. Then, 200 μL of 96% ethanol and 200 μL of PureLinkR genomic lysis/binding were added to the lysate. The lysate (about 640 μL) was vortexed briefly and loaded on the PureLinkR spin column. The spin column was centrifuged at 10, 000 × g for 1 min, and the collection tube and flow-through content were discarded. The spin column was transferred into a clean collecting tube and washed twice with appropriate wash buffers. Lastly, DNA elution was performed using 100 μL of DNA elution buffer, and the extracted DNA samples were kept at −20°C until polymerase chain reaction (PCR) analysis.

### 2.5. Detection of *Leishmania* Spp. in Dog Lymph Node DNA by PCR

The extracted DNA was subjected to PCR primers targeting the rRNA internal transcribed Spacer 2 region of *Leishmania* spp. as described by De Almeida et al. [21]. Briefly, the PCR was run in a reaction mixture of 50 μL, which included 1 μL of template DNA, 0.2 mM of each deoxynucleoside triphosphate, 0.2 μM each primer, 2.5 U of polymerase, and 1.5 mM MgCl_2_. PCR conditions were 95°C for 5 min, followed by 40 cycles of 95°C for 30 s, 60°C for 30 s, and 72°C for 1 min, and a final extension of 72°C for 7 min. The PCR included a nontemplate control (DNA/RNA free water) and *L*. *infantum* positive control DNA sample. Electrophoresis in a 1.2% agarose gel at 100 V was run to assess the PCR amplification.

## 3. Results

### 3.1. General Characteristics of the Sampled Dogs

A total of 139 dogs (88 males and 51 females) were sampled, 115 (83%) from Moroto, and 24 (17%) from Amudat District. All the sampled dogs were above 6 months. They were categorized following the country and international veterinary age protocol [19, 20], namely, juvenile (6–12 months), young adult (aged 1 year or 12–24 months), mature adult (2–6 years), senior adults (7–11 years), and geriatric (above 11 years). Demographic data, signalment (age, sex, breed, GPS, owner, and season), and dog owners' information were captured [22], and other necessary data related to dog leishmaniasis, including clinical features (Table 1). Quality control was taken to exclude dogs that had not lived at least one transmission season in the VL endemic area, and those from which the required sample volume could not be collected during the study.

### 3.2. Detection of Leishmania Antibodies by ELISA

All the ELISA test results from the 139 examined dog sera were negative for circulating *Leishmania* sp. antibodies with an OD of < 0.25, lower than the threshold value of 0.45 (https://www.diachel.gr/ingenasa-es-ingezim-leishmania-15.lsh.k.1-el). As expected from the test kit (https://www.diachel.gr/ingenasa-es-ingezim-leishmania-15.lsh.k.1-el), the positive control showed strong positivity/validity with an OD of > 0.8.

### 3.3. Detection of Leishmania DNA

**Figure 2:**
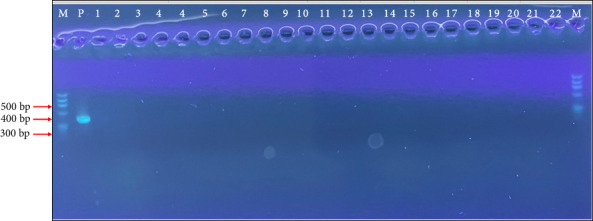
A 1.2% agarose gel obtained from PCR amplification. “M” is a 100 bp marker, “P” is the positive control *Leishmania infantum,* and “1” is a nontemplate control (DNA/RNA free water).

All the dog lymph node sample aspirates were analyzed by PCR targeting the rRNA internal transcribed Spacer 2 region, and all were negative for *Leishmania* spp.  Figure 2 shows a representative 1.2% agarose gel obtained from PCR amplification of an rRNA internal transcribed Spacer 2 region of *Leishmania* species.

## 4. Discussion

The Karamoja subregion in Uganda has reported over 100 new VL cases annually since 2013, and this is likely to worsen with the current increase in human population and changes in land use and climate if no interventions are taken. The zoonotic reservoir(s) for VL in the Karamoja subregion of Uganda have not been elucidated [23]. To our knowledge, this is the first attempt to identify VL nonhuman reservoirs within this region. We investigated the possibility that dogs are potential reservoirs for VL within the Karamoja subregion of Uganda.

Based on our findings, we did not detect any circulating *Leishmania* sp. antibodies in dog sera or *Leishmania* sp. parasites from DNA extracted from lymph node aspirates. The negative detection of *Leishmania* parasites in the studied local dogs could but attributed to one major factor; the transmission of *L*. *donovani,* responsible for the high VL cases in the Karamoja subregion, Uganda, is mostly anthroponotic, from human to human, with humans being the sole reservoirs [1, 6, 23, 24] but more interventions are currently recommended on the exact blood meal sources for the VL sand fly vectors, including *Phlebotomus orientalis*, in the study area. This is opposite for *L*. *infantum*, whose transmission is mostly zoonotic, from animals to humans, with dogs and rodents serving as reservoir hosts [1].

Previous related studies elsewhere including Machakos District, Kenya [25], Sudan [26], the Middle East (such as Yemen, United Arab Emirates, Syria, Saudi Arabia), and North African countries including Morocco, Algeria, Tunisia, Libya, Egypt [12], Suriname in South America [27], and other countries in the Old World [28] have reported dogs *C. familiaris* as the main nonhuman reservoir for VL parasites. In Machakos District, Kenya, two (0.7%) of the 288 examined dogs were VL positive with *Leishmania* amastigotes, enlarged and darkened livers and spleens [29]. In Sudan, three (6%) of the 51 examined dogs had *Leishmania* zymodemes known to be present in human VL and post-kala-azar dermal leishmaniasis cases [30]. Studies by Zijlstra and El-Hassan (2001) [31] in Sudan stated that Sudanese rodents and some carnivores serve as reservoirs of human visceral disease. Hoogstraal and Heyneman (1969) [32] detected *L*. *donovani* responsible for VL in rodents and a few wild mammals: grass rats (*Arvicanthis niloticus*), spiny mouse (*Acomys albigena*), serval *(Felis serval*), and genet (*Genetta genetta*) in Sudan, while Sixl et al. [33] also detected *L*. *donovani* in jackal (*Canis* sp.) in Sudan.

The currently recognized high VL human cases in the Karamoja subregion could be associated with socioeconomic factors such as pastoralism, the sociocultural lifestyle of living in “manyatta” houses poorly built using clay, and with holes that allow sand flies to pass in and/or through and breed. We observed several termite hills/mounds around domestic settings, kraals, and grazing grounds during the present study. The termite mounds serve as important breeding and resting sites for the sand flies in the study area. Also, the pastoralists sit and sleep on the raised termite mounds while grazing and guarding livestock; this increases the chance of human–sand fly interaction/bites. On another related risk aspect, the Karamojong environmental settings possess lots of acacia-balanites woodland and black cotton soils, which are known to be good habitats for sand flies, including *Ph*. *orientalis,* the *L*. *donovani* vector, and the causative agent for VL [34]. The detected five dogs (4%), positive for lymphadenopathy had clinical signs including enlarged/swollen lymph nodes in the jawline, neck, shoulders, and groin, deviating from the normal small, firm, and freely movable ones upon assessing by physical examinations [35]. Anemia was also assessed through clinical signs such as pale gums, pale mucous membranes (inner eyelids and inner lips), rapid heart rate, and lethargy [36]. The moderately recognized dog cachectic (29%) could be attributed to metabolic disruptions/changes, chronic infections (cancer, heart, liver, or kidney disease), and inflammation [37]. Also, the detected dog dermatitis (29%) could be attributed to flea bites, responsible for causing intense itching and inflammation, ticks and mites, known for skin infestation, causing mange, or due to allergies and/or bacterial or fungal infections [32, 33]. These clinical signs, although not indicative of canine leishmaniasis in this study, highlight the general poor health and high parasitic load in the studied dog population, which is an important context in its own right, particularly in terms of animal welfare and zoonotic disease. However, more interventions are still needed for exact cause of the recognized dog cachectic and dermatitis during the study.

### 4.1. Limitations

Our study has limitations that might have affected the results. The first one relates to the sample size of the dogs (*n* = 139); we might have likely missed positive dogs as we sampled around 5% of dogs per village. Secondly, dogs were sampled from mass vaccination campaigns instead of considering dogs from or near homesteads reporting VL cases. Thirdly, we did not sample other reservoir hosts, such as livestock, and wild animals, such as rodents known to be reservoirs for *Leishmania* parasites, nor did we monitor the status and feeding trends of the phlebotomine sand fly vectors. Future studies could benefit from sampling more dogs within VL hotspot villages and from homesteads reporting a significant number of human cases and examining the status of *Leishmania* parasites in sand flies and their feeding trends. Also, in our study, we only used ITS-2, but future related studies should use both ITS-2 and ITS-1 to aid clear parasite identification.

## 5. Conclusions

The current study has demonstrated that dogs might not be reservoirs for *L*. *donovani* in the Karamoja subregion of Uganda. Other domestic animals (such as cattle, goats, rabbits, camels, and sheep), and wild rodents could be the potential reservoirs for this disease. Studies investigating the presence of *L*. *donovani* in domestic animals, wild rodents, and sand flies and their feeding trends are required to address this knowledge gap.

## Figures and Tables

**Table 1:** Overall dog demographic and other clinical parameters during the study.

Category	Number examined (*n* = 139)
Age (months or years)	
Juvenile (6–12 months)	24 (17%)
Young adult (13–24 months)	41 (30%)
Mature adult (3–6 years)	52 (37%)
Senior adult (7–11 years)	22 (16%)
Breed	
African shepherd/local breed	132 (95%)
Unidentified breed	7 (5%)
Clinical parameters	
Fever	14 (10%)
Lymphadenopathy	5 (4%)
Cachectic	40 (29%)
Nasal or ocular lesion	2 (1%)
Dermatitis	41 (29%)
Epistaxis	2 (1%)
Anemia	14 (10%)
Lameness	5 (4%)
Cutaneous ulceration	16 (12%)

## Data Availability

The data supporting this study's findings are available from the corresponding author upon request.

## Nomenclature

VL: Visceral leishmaniasis
DO: Optical density
PCR: Polymerase chain reaction
rRNA: Ribosomal ribonucleic acid
ITS: Internal transcribed spacer
*Ph. orientalis*: *Phlebotomus orientalis*
